# Inflammatory Bowel Disease (IBD) pharmacotherapy and the risk of serious infection: a systematic review and network meta-analysis

**DOI:** 10.1186/s12876-017-0602-0

**Published:** 2017-04-14

**Authors:** Chelle L. Wheat, Cynthia W. Ko, Kindra Clark-Snustad, David Grembowski, Timothy A. Thornton, Beth Devine

**Affiliations:** 1 Department of Health Services, School of Public Health, University of Washington, 1959 NE Pacific Street, Magnuson Health Sciences Center, Room H-680, Box 357660, Seattle, WA USA; 2 Division of Gastroenterology, Department of Medicine, University of Washington, Seattle, WA USA; 3 Department of Pharmacy, School of Pharmacy, University of Washington, Seattle, WA USA; 4 Department of Biostatistics, School of Public Health, University of Washington, Seattle, WA USA

**Keywords:** Inflammatory bowel disease, IBD pharmacotherapy, Infection, Network meta-analysis

## Abstract

**Background:**

The magnitude of risk of serious infections due to available medical therapies of inflammatory bowel disease (IBD) remains controversial. We conducted a systematic review and network meta-analysis of the existing IBD literature to estimate the risk of serious infection in adult IBD patients associated with available medical therapies.

**Methods:**

Studies were identified by a literature search of PubMed, Cochrane Library, Medline, Web of Science, Scopus, EMBASE, and ProQuest Dissertations and Theses. Randomized controlled trials comparing IBD medical therapies with no restrictions on language, country of origin, or publication date were included. A network meta-analysis was used to pool direct between treatment comparisons with indirect trial evidence while preserving randomization.

**Results:**

Thirty-nine articles fulfilled the inclusion criteria; one study was excluded from the analysis due to disconnectedness. We found no evidence of increased odds of serious infection in comparisons of the different treatment strategies against each other, including combination therapy with a biologic and immunomodulator compared to biologic monotherapy. Similar results were seen in the comparisons between the newer biologics (e.g. vedolizumab) and the anti-tumor necrosis factor agents.

**Conclusions:**

No treatment strategy was found to confer a higher risk of serious infection than another, although wide confidence intervals indicate that a clinically significant difference cannot be excluded. These findings provide a better understanding of the risk of serious infection from IBD pharmacotherapy in the adult population.

**Prospero registration:**

The protocol for this systematic review was registered on PROSPERO (CRD42014013497).

**Electronic supplementary material:**

The online version of this article (doi:10.1186/s12876-017-0602-0) contains supplementary material, which is available to authorized users.

## Background

Inflammatory bowel disease (IBD) typically requires lifelong medical care for adequate disease management. Medical therapies for IBD include anti-inflammatories such as mesalamine or sulfasalazine, antibiotics, corticosteroids, immunomodulators, and biologic medications, all of which may be used alone or in combination. Each treatment strategy carries the risk of adverse effects and may not adequately manage the patient’s disease.

Corticosteroids, immunomodulators, and biologic medications in particular can have significant adverse effects, possibly including a higher risk of infection. Reactivation of latent infections, such as tuberculosis, is of specific concern with biologic medications [1]. Previous estimates of the proportion of IBD patients with any infection (not limited to serious) following treatment with these medications range from 0.5-30.0%; however there is inconsistency in the reporting of infectious outcomes in the published literature, making the true incidence of infection difficult to determine [2]. In addition, there is conflicting evidence as to whether combinations of therapies modify the risk for serious infection [3–6]. Furthermore, serious infections in particular are relatively rare, and large cohorts of treated patients are required to determine the incidence for specific medications [2]. Lastly, many of these therapies have never been compared directly to each other in the existing literature.

Understanding the risk for infections associated with IBD pharmacotherapy is a crucial consideration for providers and patients. The aim of this study is to estimate the risk of serious infection from currently available medical therapies in adult IBD patients through a systematic review and network meta-analysis of randomized controlled trials (RCTs). Unique to this study, we compare the risks of serious infection for the different IBD therapies and combinations of therapies, even in situations where medications have not been directly compared in previous studies.

## Methods

### Literature search

**Fig. 1:**
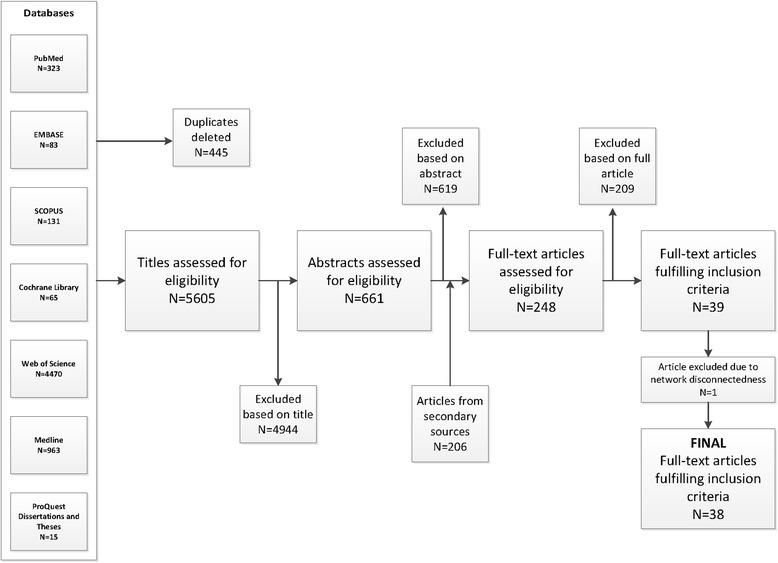
PRISMA Flowchart depicting the identification of studies, inclusion, and exclusion assessment

A detailed literature search was conducted to identify all published and unpublished RCTs of IBD pharmacotherapies in adult patients. Due to the heterogeneity in treatment and outcome reporting, observational studies (i.e. cohort, case-control) were excluded from this analysis. The classes of medications included in the search were corticosteroids (e.g. budesonide, prednisone); immunomodulators (e.g. azathioprine, 6-mercaptopurine, methotrexate); anti-inflammatories (e.g. mesalamine, sulfasalazine); antibiotics (e.g. rifaximin); and biologics (e.g. infliximab, adalimumab, certolizumab pegol, golimumab, ustekinumab, vedolizumab). We searched the PubMed, Cochrane Library, Medline, Web of Science, Scopus, EMBASE, and ProQuest Dissertations and Theses databases. Reference lists of published articles were hand searched for secondary sources, and experts in the field contacted for unpublished data. Furthermore, ClinicalTrials.gov, the WHO International Clinical Trial Registry, and scientific information packets of approved IBD pharmacotherapies were scrutinized for additional information sources. No restrictions on language, country of origin, or publication date were used. The duration of investigational treatment and follow-up were required to be at least six weeks each. The date of the final literature search was 17 March 2015. Figure 1 outlines the literature search (Additional file 1: Table S1). The protocol for this systematic review was registered on PROSPERO (CRD42014013497) and can be accessed at: http://www.crd.york.ac.uk/PROSPERO/display_record.asp?ID=CRD42014013497

### Inclusion and exclusion criteria

All RCTs that reported odds ratios (ORs) or provided information sufficient to accurately calculate ORs for serious infection in adult IBD patients were included. Serious infections were defined per the US Food and Drug Administration’s guidelines [7] as one that results in death, is life-threatening, results in hospitalization or prolongs hospitalization, causes disability or permanent damage, or is considered by the reporting investigator as an event that requires medical or surgical intervention to avoid one of these specified outcomes. Studies focusing on pediatric populations, those with incomplete reporting of serious adverse events, those without a comparison group (open-label trials), those of treatment duration and length of follow up less than 6 weeks each, and those not written in English and unable to be translated to English were excluded. If publications reported duplicate data on a population, only the publication with the longest follow-up period was included.

### Data collection and quality assessment

Two independent reviewers (CW and KCS) examined each article for inclusion according to the eligibility criteria. Any disagreement was resolved through discussion and consensus. Thirty-nine articles fulfilled the inclusion criteria (Fig. 1) [5, 8–45].

We retrieved demographic (where possible) and outcome data for each included article using standardized forms. Individual studies were assigned a bias risk rating using the Cochrane Collaboration’s Risk of Bias Assessment Tool [46]. The strength of evidence was assessed utilizing The Grades of Recommendation, Assessment, Development, and Evaluation (GRADE) approach specifically designed for network meta-analysis [47].

### Statistical analysis

A network meta-analysis (NMA) technique, also known as mixed treatment comparison methods, was used to compare the risk of serious infection associated with different medications used to treat IBD. This methodological framework allowed us to construct a network of interconnected RCTs from which we could make indirect comparisons between treatments in two trials that have one treatment in common, even in situations where treatments have not been directly compared [48–50]. For example, in trial 1 treatment A is compared to treatment B, and in trial 2 treatment B is compared to treatment C. A NMA allows us to make a valid evaluation of treatment A and treatment C although these two therapies were not directly compared in a single study. Through the use of a NMA, we were able to preserve the within trial randomized treatment comparisons, as well as add information from all of the available indirect comparisons between therapies [48–50].

The logarithm of the odds ratio (OR) for each trial and its standard error (SE) were calculated in accordance with the intention to treat principle (ITT) and used in the NMA. Each arm of the individual trials was classified according to its primary treatment strategy, and no adjustments were made for variable medication dosage. A fixed value of 0.5 was added where no events were observed in one or both groups of an individual study in order to avoid computational errors. A multivariate random-effects logistic regression model using restricted maximum likelihood (REML) was used to combine estimates. Statistical analysis was performed using Stata SE version 14 (StataCorp, College Station, TX). We performed the NMA using the *network* suite of commands published by White [51]. Graphs were generated using the published Stata routines of Chaimani [52].

A crucial consideration in any NMA is the evaluation of inconsistency, or incoherence. Indirect evidence can be combined in large samples if the assumption is made that across treatment comparisons, there are no important differences in the types of studies contributing to the comparisons, or in other words that there is consistency [53]. We assessed inconsistency using a design-by-treatment interaction model, which allows for the global testing for the presence of inconsistency in NMAs with multi-arm studies [53]. In addition to inconsistency, the transitivity assumption is important to assess in a NMA. The transitivity assumption asserts that it is equally likely that any patient in the network could have been given any of the other treatments in the network [47]. As all treatment options were randomized the transitivity assumption is satisfied. Visual assessment of a comparison-adjusted funnel plot was used to assess for the presence of publication bias and other small study effects [52]. *P*-values of ≤ .05 were considered statistically significant.

## Results

**Fig. 2:**
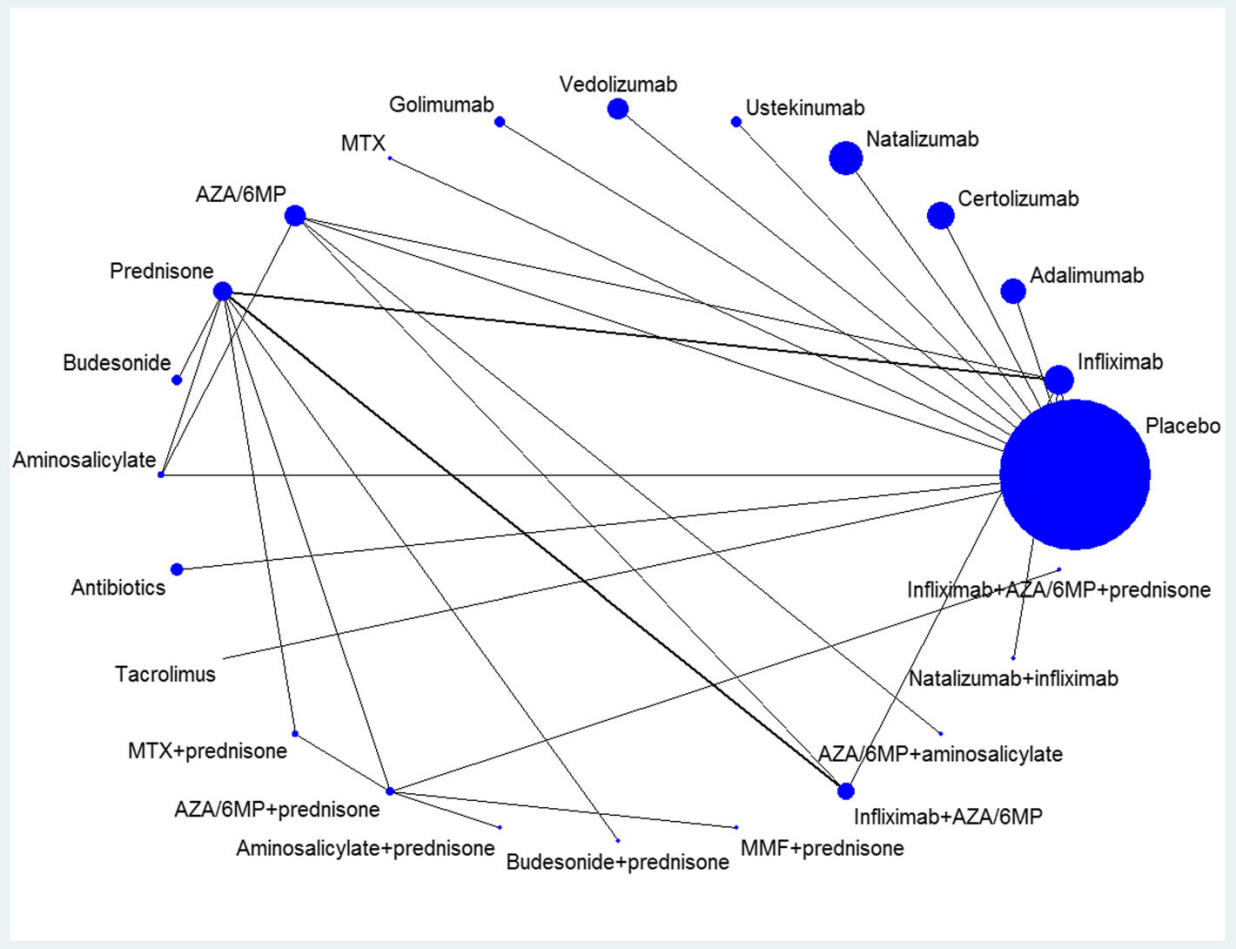
Network of clinical trials of pharmacological treatment strategies for adults with inflammatory bowel disease (IBD). Each node in the network represents a treatment strategy and the connections signify pairwise treatment comparisons from the trials included

Table 1 displays a summary of the trials included in the NMA. One of the identified trials did not fit into the connected network because of its treatment comparators (infliximab + MTX + prednisone and infliximab + prednisone), which were not examined in any of the other included trials; thus this trial was excluded and thirty-eight trials were included in the final analysis. Figure 2 illustrates the network of RCTs by treatment strategy. Each node in the network represents a treatment strategy and the connections signify pairwise treatment comparisons from the trials included. The size of the node corresponds to the number of randomly assigned participants (sample size), with a larger node signifying a larger sample size. The width of connecting lines is proportional to the number of trials comparing each pair of therapies. If there is no line connecting two nodes, no studies directly compared the two treatments [54].

**Table 1:** Characteristics of included studies

Author	Journal	Publication Year	Region of Origin	Number of Sites	Study Duration^a^ (wks)	Diagnosis	Treatment Groups	Number of Patients	Mean Age (yrs)	Female (%)
Ardizzone [8]	Dig Liver Dis	2003	Europe (Western)	1	24	CD	MTX (25 mg/week) + prednisone (40 mg/day)	27	37.0	51.9
							AZA (2 mg/kg/day) + prednisone (40 mg/day)	27	31.0	44.0
Ardizzone [9]	Gut	2006	Europe (Western)	1	24	UC	AZA (2 mg/kg/day) + prednisone (40 mg/day)	36	43.0	44.0
							5-ASA (3.2 g/day) + prednisone (40 mg/day)	36	45.0	47.0
Arora [10]	Hepatogastroenterol	1999	N America	1	52	CD	MTX (15 mg/week) + prednisone (variable)	15	37.3	20.0
							Placebo + prednisone (variable)	18	35.6	55.6
Bar-Meir [11]	Gastroenterology	1998	Middle East	14	8	CD	Budesonide (9 mg/day)	100	32.7	47.0
							Prednisone (40 mg/day)	101	32.8	49.5
Bar-Meir [12]	Dis Colon Rectum	2003	Worldwide	38	8	UC	Budesonide foam (2 mg/day)	120	42.0	62.0
							Hydrocortisone foam (100 mg/day)	128	42.0	52.0
Colombel [13]	Gastroenterology	2007	Worldwide	92	56	CD	Adalimumab (40 mg/week or 40 mg/eow)	517	NR	61.9
							Placebo	261	NR	62.1
Colombel [5]	N Engl J Med	2010	Worldwide	92	50	CD	Infliximab (5 mg/kg)	169	35.0	50.3
							AZA (2.5 mg/kg/day)	170	35.0	47.1
							Infliximab + AZA	169	34.0	47.9
Cortot [14]	Gut	2001	Worldwide	24	22	CD	Budesonide (6 mg/day) + prednisone (variable)	59	35.0	52.5
							Placebo + prednisone (variable)	58	32.0	65.6
D’Haens [15]	Lancet	2008	Europe (Western)	18	104	CD	Infliximab (5 mg/kg) + AZA (2–2.5 mg/kg/day) (or MTX)	67	30.0	66.2
							Prednisone (32 mg/day) or budesonide (9 mg/day)	66	28.7	57.8
Ewe [16]	Gastroenterology	1993	Europe (Western)	1	16	CD	AZA (2.5 mg/kg/day) + prednisone (60 mg/day)	21	27.3	NR
							Placebo + prednisone (60 mg/day)	21	29.3	NR
Feagan [17]	N Engl J Med	1995	N America	8	16	CD	MTX (25 mg/week) + prednisone (20 mg/day)	94	34.0	46.0
							Placebo + prednisone (20 mg/day)	47	36.0	45.0
Feagan [18]	N Engl J Med	2000	N America	7	40	CD	MTX (15 mg/week)	40	32.0	60.0
							Placebo	36	34.0	39.0
Feagan [19]	Gastroenterology	2014	Canada	15	50	CD	Infliximab (5 mg/kg) + MTX (25 mg/week) + prednisone (variable)	63	40.4	46.6
							Infliximab + placebo + prednisone (variable)	63	38.5	41.3
Hanauer [20]	Gastroenterology	2004	N America	5	104	CD	6MP (50 mg/day)	47	34.9	51.0
							5-ASA (3 g/day)	44	34.1	57.0
							Placebo	40	34.2	55.0
Hawthorne [21]	BMJ	1992	Europe (Western)	5	52	UC	AZA (variable)	40	50.0	62.5
							Placebo	39	40.5	33.3
Lemann [22]	Gastroenterology	2006	Europe (Western)	22	52	CD	Infliximab (5 mg/kg) + AZA/6MP (2-3 mg/kg/day or 1–1.5 mg/kg/day) + prednisone (variable)	57	26.5	52.6
							AZA/6MP + prednisone	56	27.5	57.1
Mantzaris [23]	Am J Gastroenterol	2004	Europe (Western)	1	104	UC	AZA (2.2 mg/kg/day) + 5-ASA (0.5 g TID)	36	33.0	50.0
							AZA	34	35.0	52.9
Neurath [24]	Gut	1999	Europe (Western)	1	24	CD	AZA (2.5 mg/kg/day) + prednisone (50 mg/day)	35	NR	NR
							MMF (15 mg/kg/day) + prednisone (50 mg/day)	35	NR	NR
Ochsenkun [25]	Gastroenterology	2003	Not reported	Not reported	13	UC	Infliximab (5 mg/kg)	6	NR	NR
							Prednisone (1.5 mg/kg/day)	7	NR	NR
Odonnell [26]	Gut	1992	Europe (Western)	1	6	UC	5-ASA enemas (2 g/day)	24	49.0	28.3
							Prednisone enemas (20 mg/day)	21	43.0	61.9
Oren [27]	Gastroenterology	1996	Middle East	12	36	UC	MTX (12.5 mg/day)	30	38.3	43.3
							Placebo	37	38.9	51.4
Orth [28]	Am J Gastroenterol	2000	Europe (Western)	1	52	UC	MMF (20 mg/kg/day) + prednisone (50 mg/day)	12	42.4	50.0
							AZA (2 mg/kg/day) + prednisone (50 mg/day)	12	40.4	25.0
Prantera [29]	Gastroenterology	2012	Worldwide	55	12	CD	Rifaximin (400 mg/800 mg/1200 mg BID)	308	33.3	56.8
							Placebo	102	37.0	59.0
Present [30]	N Engl J Med	1999	Worldwide	12	34	CD	Infliximab (5 mg/kg or 10 mg/kg)	63	38.1	57.1
							Placebo	31	35.4	46.0
Rutgeerts [31]	Gastroenterology	1995	Europe (Western)	1	12	CD	Metronidazole (20 mg/kg/day)	30	33.0	NR
							Placebo	30	37.0	NR
Rutgeerts [32]	N Engl J Med	2005	Worldwide	62	46	UC	Infliximab (5 mg/kg or 10 mg/kg)	243	42.1	38.3
							Placebo	121	41.4	40.5
				55	22	UC	Infliximab (5 mg/kg or 10 mg/kg)	241	40.4	40.2
							Placebo	123	39.3	42.3
Rutgeerts [33]	Gastroenterology	2005	Europe (Western)	2	54	CD	Ornidazole (1 g/day)	38	35.0	57.9
							Placebo	40	30.5	50.0
Sandborn [34]	Gastroenterology	2003	N America	18	10	CD	Tacrolimus (0.2 mg/kg/day)	21	40.8	52.4
							Placebo	25	38.1	66.0
Sandborn [35]	N Engl J Med	2005	Worldwide	142	12	CD	Natalizumab (300 mg)	724	38.0	57.0
							Placebo	181	39.0	60.0
					48	CD	Natalizumab (300 mg)	168	37.0	54.0
							Placebo	171	37.0	65.0
Sandborn [36]	Gut	2007	Worldwide	53	56	CD	Adalimumab (variable)	37	36.0	56.8
							Placebo	18	36.0	67.0
Sandborn [37]	N Engl J Med	2007	Worldwide	171	26	CD	Certolizumab pegol (400 mg)	331	37.0	53.0
							Placebo	329	38.0	60.0
Sandborn [38]	Gastroenterology	2012	Worldwide	103	52	UC	Adalimumab (variable)	248	39.6	42.7
							Placebo	246	41.3	38.2
Sandborn [39]	N Engl J Med	2012	Worldwide	153	36	CD	Ustekinumab (variable)	394	38.8	61.2
							Placebo	132	39.5	51.5
Sandborn [40]	N Engl J Med	2013	Worldwide	285	52	CD	Vedolizumab (300 mg)	967	35.7	53.4
							Placebo	148	38.6	53.4
Sandborn [41]	Gastroenterology	2014	Worldwide	251	52	UC	Golimumab (50 or 100 mg)	308	40.3	46.1
							Placebo	156	40.2	51.9
Sands [42]	Inflamm Bowel Dis	2007	N America	17	32	CD	Natalizumab (300 mg) + infliximab (5 mg/kg)	52	39.9	54.0
							Placebo + infliximab	27	38.9	37.0
Schreiber [43]	Gastroenterology	2005	Worldwide	58	20	CD	Certolizumab pegol (variable)	219	36.5	48.6
							Placebo	73	35.8	67.1
Schreiber [44]	N Engl J Med	2007	Worldwide	147	20	CD	Certolizumab pegol (400 mg)	215	38.0	57.0
							Placebo	210	38.0	48.0
Targan [45]	Gastroenterology	2007	Worldwide	114	8	CD	Natalizumab (300 mg)	259	38.1	59.0
							Placebo	250	37.7	59.0
Author	Journal	Mean Disease Duration (months)	Surgery (%)	Smoking History (%)	Concomitant Immunomodulator^b^ Use (%)	Concomitant 5-aminosalicylate Use (%)	Concomitant Steroid Use (%)	Observed Number of Serious Infections	Percentage of Serious Infections (%)	Bias Rating
Ardizzone [8]	Dig Liver Dis	76.6	33.0	NR	0.0	0.0	0.0	0	0.0	Low
		57.3	30.0	NR	0.0	0.0	0.0	0	0.0	
Ardizzone [9]	Gut	64.4	NR	25.0	0.0	0.0	0.0	0	0.0	Low
		67.5	NR	17.0	0.0	0.0	0.0	1	2.8	
Arora [1]	Hepatogastroenterol	109.2	26.7	NR	0.0	NR	0.0	1	6.7	Low
		140.4	55.6	NR	0.0	NR	0.0	0	0.0	
Bar-Meir [11]	Gastroenterology	60.0	15.0	30.0	0.0	0.0	0.0	0	0.0	Low
		60.0	23.8	31.0	0.0	0.0	0.0	0	0.0	
Bar-Meir [12]	Dis Colon Rectum	42.0	NR	41.0	0.0	52.0	0.0	0	0.0	Low
		45.6	NR	30.0	0.0	63.0	0.0	0	0.0	
Colombel [13]	Gastroenterology	NR	NR	35.6	51.0	39.3	38.9	14	2.7	Low
		NR	NR	35.6	50.6	39.5	38.7	9	3.4	
Colombel [5]	N Engl J Med	26.4	NR	NR	0.0	51.5	47.4	8	4.7	Low
		28.8	NR	NR	0.0	61.2	38.2	9	5.3	
		26.4	NR	NR	0.0	50.3	39.0	7	4.1	
Cortot [14]	Gut	106.8	30.5	NR	15.3	49.2	0.0	0	0.0	Low
		97.2	36.2	NR	8.6	48.3	0.0	0	0.0	
D’Haens [15]	Lancet	2.0	NR	55.4	0.0	4.6	0.0	4	6.0	Low
		2.5	NR	60.9	0.0	3.1	0.0	7	10.6	
Ewe [16]	Gastroenterology	55.2	NR	NR	0.0	57.0	0.0	0	0.0	Low
		46.8	NR	NR	0.0	37.0	0.0	0	0.0	
Feagan [17]	N Engl J Med	93.0	47.0	49.0	0.0	0.0	0.0	0	0.0	Low
		98.0	47.0	47.0	0.0	0.0	0.0	0	0.0	
Feagan [18]	N Engl J Med	88.0	43.0	50.0	0.0	0.0	0.0	0	0.0	Low
		84.0	36.0	42.0	0.0	0.0	0.0	1	2.8	
Feagan [19]	Gastroenterology	130.9	57.1	63.5	0.0	0.0	0.0	0	0.0	Low
		115.4	46.0	57.2	0.0	0.0	0.0	0	0.0	
Hanauer [20]	Gastroenterology	113.0	100.0	NR	0.0	0.0	0.0	0	0.0	Low
		120.0	100.0	NR	0.0	0.0	0.0	0	0.0	
		127.0	100.0	NR	0.0	0.0	0.0	0	0.0	
Hawthorne [21]	BMJ	NR	NR	NR	0.0	80.0	NR	0	0.0	Low
		NR	NR	NR	0.0	85.0	NR	0	0.0	
Lemann [22]	Gastroenterology	48.0	NR	NR	0.0	0.0	0.0	0	0.0	Low
		66.0	NR	NR	0.0	0.0	0.0	3	5.4	
Mantzaris [23]	Am J Gastroenterol	60.0	NR	8.0	0.0	0.0	0.0	0	0.0	Low
		48.0	NR	6.0	0.0	0.0	0.0	0	0.0	
Neurath [24]	Gut	NR	NR	NR	0.0	NR	0.0	0	0.0	Low
		NR	NR	NR	0.0	NR	0.0	0	0.0	
Ochsenkun [25]	Gastroenterology	NR	NR	NR	NR	NR	NR	0	0.0	Uncertain
		NR	NR	NR	NR	NR	NR	0	0.0	
Odonnell [26]	Gut	NR	NR	NR	NR	75.0	NR	0	0.0	Low
		NR	NR	NR	NR	71.4	NR	0	0.0	
Oren [27]	Gastroenterology	95.2	NR	51.7	0.0	66.7	70.0	0	0.0	Low
		70.2	NR	51.4	0.0	67.6	73.0	0	0.0	
Orth [28]	Am J Gastroenterol	149.0	0.0	NR	0.0	66.7	0.0	2	16.7	Low
		87.0	0.0	NR	0.0	66.7	0.0	1	8.3	
Prantera [29]	Gastroenterology	40.0	28.6	21.8	25.2	67.4	48.8	1	0.3	Low
		39.0	32.0	26.0	27.0	71.0	48.0	0	0.0	
Present [30]	N Engl J Med	151.2	60.3	NR	46.0	52.4	34.9	3	4.8	Low
		144.0	59.0	NR	29.0	61.0	35.0	0	0.0	
Rutgeerts [31]	Gastroenterology	108.0	NR	NR	0.0	0.0	NR	0	0.0	Low
		120.0	NR	NR	0.0	0.0	NR	0	0.0	
Rutgeerts [32]	N Engl J Med	85.8	NR	46.1	51.4	69.1	58.8	11	4.5	Low
		74.4	NR	50.4	43.8	70.2	65.3	5	4.1	
		79.2	NR	46.9	42.3	75.9	52.3	5	2.1	
		78.0	NR	48.8	43.9	72.4	48.8	1	0.8	
Rutgeerts [33]	Gastroenterology	84.0	100.0	44.7	0.0	0.0	52.3	0	0.0	Low
		36.0	100.0	47.5	0.0	0.0	35.0	0	0.0	
Sandborn [34]	Gastroenterology	NR	62.0	33.0	62.0	43.0	24.0	0	0.0	Low
		NR	44.0	16.0	14.0	40.0	16.0	0	0.0	
Sandborn [35]	N Engl J Med	121.0	41.0	23.0	34.0	47.0	38.0	12	1.7	Low
		110.0	40.0	24.0	28.0	44.0	40.0	4	2.2	
		119.0	33.0	16.0	37.0	9.0	38.0	6	3.6	
		116.0	40.0	26.0	35.0	54.0	46.0	5	2.9	
Sandborn [36]	Gut	101.2	NR	86.5	24.3	70.3	45.9	0	0.0	Low
		98.9	NR	67.0	18.0	44.0	56.0	0	0.0	
Sandborn [37]	N Engl J Med	84.0	36.0	31.0	21.0	NR	22.0	7	2.1	Low
		96.0	34.0	33.0	20.0	NR	23.0	3	0.9	
Sandborn [38]	Gastroenterology	97.2	NR	NR	37.5	58.9	60.5	4	1.6	Low
		102.0	NR	NR	32.5	63.0	56.9	5	2.0	
Sandborn [39]	N Engl J Med	147.6	NR	NR	24.4	17.0	48.0	10	2.5	Low
		148.8	NR	NR	22.7	18.2	55.3	8	6.1	
Sandborn [40]	N Engl J Med	110.4	42.6	27.3	16.1	NR	34.7	45	4.7	Low
		98.4	36.5	23.0	16.9	NR	30.4	9	6.1	
Sandborn [41]	Gastroenterology	84.0	NR	NR	30.8	80.2	53.8	10	3.2	Low
		82.8	NR	NR	33.3	80.1	56.4	3	1.9	
Sands [42]	Inflamm Bowel Dis	150.3	NR	NR	50.0	46.0	27.0	0	0.0	Low
		120.0	NR	NR	56.0	37.0	30.0	0	0.0	
Schreiber [43]	Gastroenterology	99.6	36.1	NR	37.4	44.3	34.7	1	0.4	Low
		95.4	37.0	NR	35.6	39.7	39.7	0	0.0	
Schreiber [44]	N Engl J Med	108.0	30.0	30.0	27.0	NR	22.0	6	2.8	Low
		84.0	35.0	36.0	25.0	NR	21.0	2	0.9	
Targan [45]	Gastroenterology	121.4	NR	NR	37.0	49.0	42.0	1	0.4	Low
		120.3	NR	NR	38.0	48.0	38.0	4	1.6	

*Abbreviations*: *CD* Crohn’s Disease, *UC* ulcerative colitis, *NR* not reported, *MTX* methotrexate, *AZA* azathioprine, *6MP* 6-mercaptopurine, *MMF* mycophenolate mofetil

^a^inclusive of active treatment period and follow-up

^b^includes MTX, 6MP, AZA

&Step-up paradigm starting with prednisone then progressing to AZA

Table 2 provides the estimated odds of serious infection for all treatment strategies compared to placebo. Amongst all therapy contrasts, no statistically significantly increased odds of serious infection were discovered. However, the confidence intervals were extremely wide in many of the comparisons, and a clinically significant increase in infection risk could not be excluded. (Additional file 2: Table S2). Table 3 displays the estimates for selected therapeutic strategies compared to the anti-tumor necrosis factor (anti-TNF) biologics including infliximab, adalimumab, and certolizumab pegol. In the comparison of ustekinumab with certolizumab pegol there was found to be a lower odds of serious infection (OR 0.17, 95% CI 0.04–0.67). No statistically significant increased odds of serious infection were observed for any other treatment comparisons including those between the specific anti-TNFs agents (i.e. adalimumab vs. infliximab), as well as those between anti-TNF monotherapy and dual therapy with an immunomodulator (i.e. infliximab alone vs. infliximab + azathioprine/6MP) (Additional file 3: Table S3).

**Table 2:** Estimated odds of serious infection for treatment strategies compared to placebo

Treatment strategy	Comparator	Odds ratio	Standard error	95% Confidence interval	
Infliximab	Placebo	1.36	0.45	0.57	3.27
Adalimumab	Placebo	0.77	0.36	0.38	1.56
Certolizumab pegol	Placebo	2.37	0.50	0.88	6.38
Natalizumab	Placebo	0.80	0.40	0.37	1.73
Ustekinumab	Placebo	0.40	0.49	0.16	1.05
Vedolizumab	Placebo	0.75	0.38	0.36	1.58
Golimumab	Placebo	1.71	0.67	0.46	6.31
Methotrexate	Placebo	0.52	1.28	0.04	6.34
Azathioprine/6MP	Placebo	1.43	0.61	0.43	4.76
Prednisone	Placebo	1.92	0.85	0.36	10.21
Budesonide	Placebo	1.99	1.65	0.08	50.97
Aminosalicylate	Placebo	1.37	1.40	0.09	21.47
Antibiotic	Placebo	1.01	1.07	0.12	8.34
Tacrolimus	Placebo	1.19	2.02	0.02	62.32
Methotrexate + prednisone	Placebo	2.94	1.45	0.17	50.23
Azathioprine/6MP + prednisone	Placebo	2.37	1.76	0.07	75.25
Aminosalicylate + prednisone	Placebo	7.32	2.41	0.06	832.34
Budesonide + prednisone	Placebo	1.89	2.18	0.03	135.88
MMF + prednisone	Placebo	4.14	2.07	0.07	241.50
Infliximab + azathioprine/6MP	Placebo	1.10	0.65	0.31	3.97
Azathioprine/6MP + aminosalicylate	Placebo	1.35	2.11	0.02	83.66
Natalizumab + inflximab	Placebo	0.71	2.06	0.01	40.65
Infliximab + azathioprine/6MP + prednisone	Placebo	0.32	2.33	0.00	30.40

*Abbreviations*: *6MP* 6-mercaptopurine, *MMF* mycophenolate mofetil

**Table 3:** Estimated odds of serious infection for selected^a^ treatment strategies compared to anti-tumor necrosis factor biologics

Treatment strategy	Comparator	Odds ratio	Standard error	95% Confidence interval	
Adalimumab	Infliximab	0.57	0.57	0.18	1.74
Certolizumab pegol	Infliximab	1.74	0.67	0.47	6.53
Natalizumab	Infliximab	0.58	0.60	0.18	1.88
Ustekinumab	Infliximab	0.30	0.66	0.08	1.08
Vedolizumab	Infliximab	0.55	0.58	0.18	1.74
Golimumab	Infliximab	1.26	0.80	0.26	6.05
Infliximab + azathioprine/6MP	Infliximab	0.81	0.50	0.30	2.17
Infliximab + azathioprine/6MP + prednisone	Infliximab	0.23	2.29	0.00	20.82
Certolizumab pegol	Adalimumab	3.08	0.62	0.91	10.37
Natalizumab	Adalimumab	1.03	0.54	0.36	2.94
Ustekinumab	Adalimumab	0.52	0.60	0.16	1.71
Vedolizumab	Adalimumab	0.98	0.52	0.35	2.71
Golimumab	Adalimumab	2.22	0.76	0.50	9.78
Natalizumab	Certolizumab pegol	0.34	0.64	0.10	1.18
Ustekinumab	Certolizumab pegol	0.17	0.70	0.04	0.67
Vedolizumab	Certolizumab pegol	0.32	0.63	0.09	1.09
Golimumab	Certolizumab pegol	0.72	0.84	0.14	3.71

*Abbreviations*: *6MP* 6-mercaptopurine, *MMF* mycophenolate mofetil

^a^Other group comparisons can be found in Additional file 3: Table S3

Furthermore, no statistically significant increased odds of serious infection were found for any comparison in contrasting each therapy with the immunomodulators (azathioprine/6MP and methotrexate) or other commonly used therapies such as prednisone, budesonide, and tacrolimus (Table 4; Additional file 4: Table S4 and Additional file 5: Table S5). Similar findings were seen for the newer biologic pharmacotherapies including natalizumab, ustekinumab, and vedolizumab (Table 4; Additional file 6: Table S6). Lastly, no increased odds of serious of infection were found in comparisons of each included treatment strategy against other combinations of therapies such as methotrexate/prednisone or azathioprine/6MP + prednisone (Additional file 7: Table S7). However again, the confidence intervals were extremely wide in many of the comparisons, and a clinically significant increase in infection risk could not be excluded.

**Table 4:** Estimated odds of serious infection for selected^a^ treatment strategies of interest

Treatment strategy	Comparator	Odds ratio	Standard error	95% Confidence interval	
Azathioprine/6MP	Methotrexate	2.75	1.42	0.17	44.07
Prednisone	Azathioprine/6MP	1.34	0.76	0.30	5.96
Infliximab + azathioprine/6MP	Azathioprine/6MP	0.77	0.50	0.29	2.06
Ustekinumab	Natalizumab	0.51	0.63	0.15	1.73
Vedolizumab	Natalizumab	0.95	0.55	0.32	2.76
Golimumab	Natalizumab	2.15	0.77	0.47	9.82
Vedolizumab	Ustekinumab	1.87	0.61	0.56	6.22
Golimumab	Ustekinumab	4.24	0.82	0.84	21.32
Golimumab	Vedolizumab	2.27	0.76	0.51	10.16

*Abbreviations*: *6MP* 6-mercaptopurine

^a^Other group comparisons can be found in Additional files 1, 2, 3, 4, 5, 6, and 7

In the design-by-treatment interaction model, no evidence of inconsistency was found (chi^2 = 0.25, *p* = .99). Visual assessment of a comparison-adjusted funnel plot did not reveal any evidence of publication bias or other small study effects. Although the included studies were randomized, there were low rates of completed follow-up as well as selective cross over amongst therapy groups in many trials contributing to a high risk of bias. In addition, although the transitivity assumption was satisfied through randomization, there may remain differences in the study populations that modify the effect, thus, the overall quality of the body of evidence per the GRADE approach is low.

## Discussion

In this network meta-analysis (NMA), we combined clinical trial data from thirty-eight published articles that included twenty-four different treatment strategies for IBD. These results summarize the risk of serious infection from available RCTs of commonly prescribed IBD pharmacotherapies. The study overcomes some of the limitations from previous studies by applying a universal definition for serious infection and examining large cohorts of treated patients from RCTs. Furthermore, the NMA technique allows for investigation of multiple therapies, including combinations of therapies, which have not been previously compared directly.

Our results show that no treatment strategy exhibits a higher odds of serious infection than another (including placebo), although in many cases the confidence intervals were wide, likely due to the small number of studies examining specific therapies available, and thus did not exclude a clinically significant increase in risk. Of particular interest, patients treated with dual immunosuppression with biologic medications and immunomodulators do not appear to be at higher risk of serious infection compared to those treated with biologic monotherapy, at least in the short-term. This lends additional support towards the safety of combination therapy as a viable treatment strategy, especially for those patients who are at high risk of antibody formation and subsequent loss of response from some biologic therapies. Our results are in contrast to those reported by Toruner et al. who showed an increased risk of opportunistic infections among patients treated with combination therapy in a retrospective case–control study [4]. This discrepancy is likely due to differences in study design and patient populations, as our meta-analysis is limited to patients enrolled in RCTs. First, the Toruner study could not assess disease severity; thus medication use could be a marker for disease severity rather than a true risk factor for infection. Second, patients who are eligible to be enrolled in an RCT are extensively screened for infection and other comorbidities prior to enrollment and followed more closely than in clinical practice. It is possible that this additional scrutiny both selected out patients who were more prone to infection and/or modified their risk for development of infection sometime during the trial period. Furthermore, the patients in the Toruner study were from one academic medical center and results may not be generalizable to other settings.

We found no evidence of a higher odds of serious infection from the newly available biologic therapies, such as vedolizumab and ustekinumab, compared to the anti-tumor necrosis factor (anti-TNF) biologic agents (or to one another). Given the growing number of patients who have lost response or who are intolerant to anti-TNFs, these findings are reassuring. Furthermore, we specifically looked at the comparison of those on triple immunosuppression (i.e. biologic + immunomodulator + steroid) versus those on combination therapy or biologic monotherapy, and similar results were found. Of note, we did observe a trend towards increased risk for serious infection with prednisone treatment, either as monotherapy or combined with other therapies. This trend was not statistically significant in any of the comparisons; however it is consistent with the existing evidence of the association of prolonged corticosteroid use and infection [4].

Previous estimates of the risk of serious infection related to IBD therapy vary widely, and the absolute risk is difficult to quantify. The SONIC trial assessed the relative risk of serious infection from azathioprine alone, infliximab alone, and azathioprine and infliximab together (combination therapy), and the authors found no statistically significant differences among the groups [5]. In addition, the absolute risk of serious infection was low in each group: azathioprine (5.6%), infliximab (4.9%), and combination therapy (3.9%) over a mean follow-up of 125.7 patient-years [5]. In contrast, a case control study at the Mayo Clinic found an increased risk of serious infection from combination therapy compared to infliximab monotherapy, as well as an increased odds of serious infection from infliximab, corticosteroids, and azathioprine/6MP alone compared to no medication [4]. Although these two well-known studies have conflicting results, the differences have been explained as likely due to the different patient populations [55]. Crohn’s disease (CD) is associated with more disease related infectious complications (e.g. abscesses) than ulcerative colitis (UC), but it is unclear if the risk of infectious complications differs between CD and UC. Our study, like many, did not examine CD and UC separately as we combined the two conditions in order to maximize the number of patients available for the analysis. In addition, the two conditions are often difficult to distinguish and there is significant overlap between the treatment paradigms.

In a recently published update to the ENCORE registry study, D’Haens et al. report an increased risk of serious infection among CD patients treated with infliximab compared to other IBD therapies [56]. Although the relative risk of serious infection was higher among those treated with infliximab or combination therapy, the absolute risk of infection remained low. The differences again are likely due to differing patient populations and our examination of CD and UC combined. Our results are comparable to the existing literature, in particular the SONIC trial, and suggest that the shorter-term risk of serious infection from IBD pharmacotherapy is low.

Our findings do have some limitations. First, we only included data from RCTs due to the added heterogeneity non-randomized studies would contribute to the analysis, as well as the desire to preserve the benefits of randomization in our analysis. This limits the external validity and representativeness of our findings, especially given the strict entry criteria for these trials. Patients with comorbidities or other characteristics excluded from these studies may be at higher risk of serious infections from IBD pharmacotherapy than those included in our study populations. Second, RCTs are not specifically designed or powered to investigate adverse events such as serious infections; thus we may underestimate the true association of these therapies with serious infection, a limitation that is not overcome by pooling evidence in a meta-analysis. Third, there was variable length of treatment and follow up among the included studies, which may underestimate the risk of serious infection. Many of the included trials had short follow-up duration and did not include details on the time to development of the infections, so longer-term risk of these therapies were not quantified. In a recently published article, the median time to the development of active tuberculosis after initiation of an anti-TNF was approximately three months, which lends support to the possibility that the true risk of serious infection could be underestimated in published RCTs; however the average duration of the RCTs included in this meta-analysis was 37 weeks, thus we expect that the included studies would have detected a large proportion of cases of tuberculosis [57]. However, this does not exclude the possibility that other serious infections with longer time to development were underreported. Fourth, the direct estimates for some therapeutic strategies are based on a single study due to the lack of available trial data. Fifth, RCTs investigating the newer biologics (e.g. vedolizumab, ustekinumab) have been published since the last literature search and are not included in this meta-analysis, which may influence our results. Lastly, traditional limitations of meta-analyses due to variations in the treatment regimens, in the study populations, and in the conduct of the individual trials may bias our estimates, the direction of which is indeterminable.

Despite these limitations, this study provides crucial information regarding one of the most clinically significant risks of interest associated with IBD pharmacotherapy. Our findings are robust in terms of the low estimate of inconsistency for our model and the completeness of the literature search of studies for inclusion. As additional data becomes available regarding IBD therapies, this information can be added to the network to increase our confidence in the estimates.

## Conclusions

Our results add to the body of evidence regarding risks and benefits of IBD pharmacotherapy, and suggest that commonly used therapies are not associated with increased risk of serious infections in the first several months of treatment, although confidence intervals were wide for many comparisons; thus a clinically significant difference cannot be excluded. Further, long-term studies using larger cohorts will supplement these findings and increase the generalizability of these results.

## Additional files

Additional file 1: Table S1. Search Algorithms. (DOCX 30 kb)

Additional file 2: Table S2. Estimated odds of serious infection for treatment strategies compared to aminosalicylates or antibiotics. (DOCX 29 kb)

Additional file 3: Table S3. Estimated odds of serious infection for treatment strategies compared to anti-tumor necrosis factor biologics. (DOCX 38 kb)

Additional file 4: Table S4. Estimated odds of serious infection for treatment strategies compared to Immunomodulators. (DOCX 30 kb)

Additional file 5: Table S5. Estimated odds of serious infection for treatment strategies compared to other immunosuppressants. (DOCX 33 kb)

Additional file 6: Table S6. Estimated odds of serious infection for treatment strategies compared to other biologics. (DOCX 35 kb)

Additional file 7: Table S7. Estimated odds of serious infection for treatment strategies compared to combination therapies. (DOCX 33 kb)

## Abbreviations

IBD: Inflammatory bowel disease
NMA: Network meta-analysis
RCT: Randomized controlled trial
WHO: World Health Organization
OR: Odds ratio
SE: Standard error
MTX: Methotrexate
6MP: 6-mercaptopurine
anti-TNF: Anti-tumor necrosis factor
PRISMA: Preferred Reporting Items for Systematic Reviews and Meta-Analyses
