# Supplementary material for: Inflammatory Bowel Disease (IBD) pharmacotherapy and the risk of serious infection: a systematic review and network meta-analysis
Source: BMC Gastroenterol. 2017 Apr 14;17:52. doi: 10.1186/s12876-017-0602-0 (PMC5391579; doi:10.1186/s12876-017-0602-0)
Supplement: Supplementary file 1 — Search Algorithms. (DOCX 30 kb) [file 12876_2017_602_MOESM1_ESM.docx]

Supplementary Table 1: Search Algorithms

| **PUBMED (results returned=323)** |
| --- |
| (((((((((((((inflammatory bowel diseases[MeSH Terms]) OR inflammatory bowel disease[Title/Abstract]) OR inflammatory bowel diseases[Title/Abstract]) OR crohn's disease[Title/Abstract]) OR crohns disease[Title/Abstract]) OR crohn disease[Title/Abstract]) OR ulcerative colitis[Title/Abstract]) OR colitis[Title/Abstract]) OR enterocolitis[Title/Abstract]) OR ileocolitis[Title/Abstract]) OR ileitis[Title/Abstract])) AND ((((((((((((((((((((((((((((((((((((((((((((((((((((((((((((((((((((Anti-bacterial agents[MeSH Terms]) OR Tumor Necrosis Factor-alpha[MeSH Terms]) OR Integrin alpha4[MeSH Terms]) OR Antibodies, monoclonal[MeSH Terms]) OR Gastrointestinal agents[MeSH Terms]) OR Aminosalicylic acids[MeSH Terms]) OR Mesalamine[MeSH Terms]) OR Anti-inflammatory agents[MeSH Terms]) OR Methylprednisolone hemisuccinate[MeSH Terms]) OR Budesonide[MeSH Terms]) OR Adrenal cortex hormones[MeSH Terms]) OR Azathioprine[MeSH Terms]) OR 6-mercaptopurine[MeSH Terms]) OR Methotrexate[MeSH Terms]) OR Antimetabolites[MeSH Terms]) OR Cyclosporine[MeSH Terms] OR Antibacterial agents[Title/Abstract]) OR Anti-tumor necrosis factor[Title/Abstract]) OR Simponi[Title/Abstract]) OR Golimumab[Title/Abstract]) OR Hu65mira[Title/Abstract]) OR Adalimumab[Title/Abstract]) OR Cimzia[Title/Abstract]) OR Certolizumab pegol[Title/Abstract]) OR Remicade[Title/Abstract]) OR Infliximab[Title/Abstract]) OR Anti-TNF[Title/Abstract]) OR Stelara[Title/Abstract]) OR Ustekinumab[Title/Abstract]) OR Vedolizumab[Title/Abstract]) OR Tysabri[Title/Abstract]) OR Natalizumab[Title/Abstract]) OR Monoclonal antibodies[Title/Abstract]) OR Balsalizide[Title/Abstract]) OR Olsalazine[Title/Abstract]) OR Rowasa[Title/Abstract]) OR Canasa[Title/Abstract]) OR Apriso[Title/Abstract]) OR Lialda[Title/Abstract]) OR Colazal[Title/Abstract]) OR Dipentum[Title/Abstract]) OR Salofalk[Title/Abstract]) OR Pentasa[Title/Abstract]) OR Asacol[Title/Abstract]) OR Sulfasalazine[Title/Abstract]) OR Azulfidine[Title/Abstract]) OR 5-aminosalicylates[Title/Abstract]) OR aminosalicylate AND (s) AND title/abstract) OR 5-ASA[Title/Abstract]) OR Mesalamine[Title/Abstract]) OR Solumedrol[Title/Abstract]) OR Methylprednisolone hemisuccinate[Title/Abstract]) OR Budesonide[Title/Abstract]) OR Entocort[Title/Abstract]) OR Prednisone[Title/Abstract]) OR Corticosteroids[Title/Abstract]) OR Imuran[Title/Abstract]) OR Azathioprine[Title/Abstract]) OR Purinethol[Title/Abstract]) OR 6MP[Title/Abstract]) OR 6-mercaptopurine[Title/Abstract]) OR Amethopterin[Title/Abstract]) OR Methotrexate[Title/Abstract]) OR Antimetabolites[Title/Abstract]) OR Cellcept[Title/Abstract]) OR MMF[Title/Abstract]) OR Mycophenolate mofetil[Title/Abstract]) OR Cyclosporine[Title/Abstract]) OR Antibiotics[Title/Abstract]))) AND ((((((((((("Bacterial Infections[MeSH Terms] AND Mycoses"[MeSH Terms])) OR Virus Diseases[MeSH Terms]) OR Bacterial infection[Title/Abstract]) OR Coccidioidmycosis[Title/Abstract]) OR Histoplasmosis[Title/Abstract]) OR Tuberculosis[Title/Abstract]) OR Viral infection[Title/Abstract]) OR Viral diseases[Title/Abstract]) OR Fungal infection[Title/Abstract]) OR Mycoses[Title/Abstract]) |

| **EMBASE (results returned=83)** |
| --- |
| 'inflammatory bowel diseases':ab,ti OR 'crohns disease':ab,ti OR colitis:ab,ti OR enterocolitis:ab,ti OR ileitis:ab,ti OR ileocolitis:ab,ti AND (antibacterial AND agents:ab,ti OR antibiotics:ab,ti OR mycophenolate AND mofetil:ab,ti OR mmf:ab,ti OR cellcept:ab,ti OR antimetabolites:ab,ti OR methotrexate:ab,ti OR amethopterin:ab,ti OR '6 mercaptopurine':ab,ti OR mercaptopurine:ab,ti OR purinethol:ab,ti OR 6mp:ab,ti OR azathioprine:ab,ti OR adrenal AND cortex AND hormones:ab,ti OR corticosteroids:ab,ti OR budesonide:ab,ti OR entocort:ab,ti OR methylprednisolone AND hemisuccinate:ab,ti OR solumedrol:ab,ti OR 'anti inflammatory' AND agents:ab,ti OR mesalamine:ab,ti OR aminosalicylic AND acids:ab,ti OR gastrointestinal AND agents:ab,ti OR 'gastrointestinal agents' OR '5 aminosalicylate':ab,ti OR azulfidine:ab,ti OR sulfasalazine:ab,ti OR asacol:ab,ti OR pentasa:ab,ti OR salofalk:ab,ti OR dipentum:ab,ti OR colazal:ab,ti OR lialda:ab,ti OR apriso:ab,ti OR canasa:ab,ti OR rowasa:ab,ti OR olsalazine:ab,ti OR balsalizide:ab,ti OR antibodies, AND monoclonal:ab,ti OR monoclonal AND antibodies:ab,ti OR natalizumab:ab,ti OR tysabri:ab,ti OR vedolizumab:ab,ti OR ustekinumab:ab,ti OR stelara:ab,ti OR integrin AND alpha4:ab,ti OR tumor AND necrosis AND factor AND alpha:ab,ti OR 'anti-tnf' OR infliximab:ab,ti OR remicade:ab,ti OR certolizumab AND pegol:ab,ti OR cimzia:ab,ti OR adalimumab:ab,ti OR humira:ab,ti OR golimumab:ab,ti OR simponi:ab,ti OR cyclosporine:ab,ti OR 'anti-tumor necrosis factor') AND ('bacterial infection':ab,ti OR 'mycoses':ab,ti OR 'fungal infection':ab,ti OR 'viral diseases':ab,ti OR 'viral infection':ab,ti OR 'tuberculosis':ab,ti OR 'histoplasmosis':ab,ti OR 'coccidioidmycosis':ab,ti) AND [embase]/lim |
| **SCOPUS (results returned=131)** |
| ALL(inflammatory bowel disease* OR crohn* disease OR colitis OR enterocolitis OR ileitis OR ileocolitis) AND ALL(antibiotics OR mycophenolate mofetil OR MMF OR cellcept OR methotrexate OR amethopterin OR 6-mercaptopurine OR mercaptopurine OR 6MP OR purinethol OR azathioprine OR corticosteroids OR budesonide OR entocort OR methylprednisolone hemisuccinate OR solumedrol OR mesalamine OR aminosalicylic acids OR 5-aminosalicylate OR azulfidine OR sulfasalazine OR asacol OR pentasa OR salofalk OR dipentum OR colazal OR lialda OR apriso OR canasa OR rowasa OR olsalazine OR balsalizide OR natalizumab OR tysabri OR vedolizumab OR ustekinumab OR stelara OR infliximab OR remicade OR certolizumab pegol OR cimzia OR adalimumab OR humira OR golimumab OR simponi OR cyclosporine) AND ALL(bacterial infection OR mycoses OR fungal infection OR viral diseases OR viral infection OR tuberculosis OR histoplasmosis OR coccidioidmycosis) |
| **COCHRANE LIBRARY (results returned=65)** |
| inflammatory bowel disease* OR crohn* disease OR ulcerative colitis OR colitis OR enterocolitis OR ileitis OR ileocolitis in Title, Abstract, Keywords and antibacterial agents OR antibiotics OR mycophenolate mofetil OR MMF OR cellcept OR antimetabolites OR methotrexate OR amethopterin OR 6-mercaptopurine OR mercaptopurine OR 6MP OR purinethol OR azathioprine OR adrenal cortex hormones OR corticosteroids OR budesonide OR entocort OR methylprednisolone hemisuccinate OR solumedrol OR anti-inflammatory agents OR mesalamine OR aminosalicylic acids OR gastrointestinal agents OR 5-aminosalicylate OR azulfidine OR sulfasalazine OR asacol OR pentasa OR salofalk OR dipentum OR colazal OR lialda OR apriso OR canasa OR rowasa OR olsalazine OR balsalizide OR antibodies, monoclonal OR monoclonal antibodies OR natalizumab OR tysabri OR vedolizumab OR ustekinumab OR stelara OR integrin alpha4 OR tumor necrosis factor alpha OR anti-TNF OR infliximab OR remicade OR certolizumab pegol OR cimzia OR adalimumab OR humira OR golimumab OR simponi OR anti-tumor necrosis factor OR cyclosporine in Title, Abstract, Keywords and bacterial infection OR mycoses OR fungal infection OR viral diseases OR viral infection OR tuberculosis OR histoplasmosis OR coccidioidmycosis in Title, Abstract, Keywords (Word variations have been searched) |
| **WEB OF SCIENCE (results returned=4470)** |
| TS=(inflammatory bowel disease* OR crohn* disease OR ulcerative colitis OR colitis OR enterocolitis OR ileitis OR ileocolitis) AND TS=(antibacterial agents OR antibiotics OR mycophenolate mofetil OR MMF OR cellcept OR antimetabolites OR methotrexate OR amethopterin OR 6-mercaptopurine OR mercaptopurine OR 6MP OR purinethol OR azathioprine OR adrenal cortex hormones OR corticosteroids OR budesonide OR entocort OR methylprednisolone hemisuccinate OR solumedrol OR anti-inflammatory agents OR mesalamine OR aminosalicylic acids OR gastrointestinal agents OR 5-aminosalicylate OR azulfidine OR sulfasalazine OR asacol OR pentasa OR salofalk OR dipentum OR colazal OR lialda OR apriso OR canasa OR rowasa OR olsalazine OR balsalizide OR antibodies, monoclonal OR monoclonal antibodies OR natalizumab OR tysabri OR vedolizumab OR ustekinumab OR stelara OR integrin alpha4 OR tumor necrosis factor alpha OR anti-TNF OR infliximab OR remicade OR certolizumab pegol OR cimzia OR adalimumab OR humira OR golimumab OR simponi OR anti-tumor necrosis factor OR cyclosporine) AND  TS=(bacterial infection* OR mycoses OR fungal infection* OR viral disease* OR viral infection* OR tuberculosis OR histoplasmosis OR coccidioidmycosis) |
| **MEDLINE (results returned=963)** |
| TX(inflammatory bowel disease* OR crohn* disease OR ulcerative colitis OR colitis OR enterocolitis OR ileitis OR ileocolitis) AND TX(antibacterial agents OR antibiotics OR mycophenolate mofetil OR MMF OR cellcept OR antimetabolites OR methotrexate OR amethopterin OR 6-mercaptopurine OR mercaptopurine OR 6MP OR purinethol OR azathioprine OR adrenal cortex hormones OR corticosteroids OR budesonide OR entocort OR methylprednisolone hemisuccinate OR solumedrol OR anti-inflammatory agents OR mesalamine OR aminosalicylic acids OR gastrointestinal agents OR 5-aminosalicylate OR azulfidine OR sulfasalazine OR asacol OR pentasa OR salofalk OR dipentum OR colazal OR lialda OR apriso OR canasa OR rowasa OR olsalazine OR balsalizide OR antibodies, monoclonal OR monoclonal antibodies OR natalizumab OR tysabri OR vedolizumab OR ustekinumab OR stelara OR integrin alpha4 OR tumor necrosis factor alpha OR anti-TNF OR infliximab OR remicade OR certolizumab pegol OR cimzia OR adalimumab OR humira OR golimumab OR simponi OR anti-tumor necrosis factor OR cyclosporine) AND TX(bacterial infection* OR mycoses OR fungal infection* OR viral disease* OR viral infection* OR tuberculosis OR histoplasmosis OR coccidioidmycosis) |
| **ProQuest Dissertations and Theses (results returned=15)** |
| all(inflammatory bowel disease* OR crohn* disease OR ulcerative colitis OR colitis OR enterocolitis OR ileitis OR ileocolitis) AND all(antibacterial agents OR antibiotics OR mycophenolate mofetil OR MMF OR cellcept OR antimetabolites OR methotrexate OR amethopterin OR 6-mercaptopurine OR mercaptopurine OR 6MP OR purinethol OR azathioprine OR adrenal cortex hormones OR corticosteroids OR budesonide OR entocort OR methylprednisolone hemisuccinate OR solumedrol OR anti-inflammatory agents OR mesalamine OR aminosalicylic acids OR gastrointestinal agents OR 5-aminosalicylate OR azulfidine OR sulfasalazine OR asacol OR pentasa OR salofalk OR dipentum OR colazal OR lialda OR apriso OR canasa OR rowasa OR olsalazine OR balsalizide OR antibodies, monoclonal OR monoclonal antibodies OR natalizumab OR tysabri OR vedolizumab OR ustekinumab OR stelara OR integrin alpha4 OR tumor necrosis factor alpha OR anti-TNF OR infliximab OR remicade OR certolizumab pegol OR cimzia OR adalimumab OR humira OR golimumab OR simponi OR anti-tumor necrosis factor OR cyclosporine) AND all(bacterial infection* OR mycoses OR fungal infection* OR viral disease* OR viral infection* OR tuberculosis OR histoplasmosis OR coccidioidomycosis) |
