# Supplementary material for: Inflammatory Bowel Disease (IBD) pharmacotherapy and the risk of serious infection: a systematic review and network meta-analysis
Source: BMC Gastroenterol. 2017 Apr 14;17:52. doi: 10.1186/s12876-017-0602-0 (PMC5391579; doi:10.1186/s12876-017-0602-0)
Supplement: Supplementary file 2 — Estimated odds of serious infection for treatment strategies compared to aminosalicylates or antibiotics. (DOCX 29 kb) [file 12876_2017_602_MOESM2_ESM.docx]

Supplementary Table 2: Estimated odds of serious infection for treatment strategies compared to aminosalicylates or antibiotics

| **Treatment Strategy** | **Comparator** | **Odds Ratio** | **Standard Error** | **95% Confidence Interval** | |
| --- | --- | --- | --- | --- | --- |
| Antibiotic | Aminosalicylate | 0.74 | 1.77 | 0.02 | 23.62 |
| Tacrolimus | Aminosalicylate | 0.86 | 2.46 | 0.01 | 107.46 |
| Methotrexate+prednisone | Aminosalicylate | 2.14 | 1.82 | 0.06 | 75.19 |
| Azathioprine/6MP+prednisone | Aminosalicylate | 1.73 | 2.08 | 0.03 | 101.16 |
| Aminosalicylate+prednisone | Aminosalicylate | 5.34 | 2.65 | 0.03 | 964.69 |
| Budesonide+prednisone | Aminosalicylate | 1.38 | 2.44 | 0.01 | 164.60 |
| MMF+prednisone | Aminosalicylate | 3.02 | 2.35 | 0.03 | 299.54 |
| Infliximab+azathioprine/6MP | Aminosalicylate | 0.80 | 1.38 | 0.05 | 12.00 |
| Azathioprine/6MP+aminosalicylate | Aminosalicylate | 0.98 | 2.44 | 0.01 | 117.79 |
| Natalizumab+inflximab | Aminosalicylate | 0.52 | 2.44 | 0.00 | 62.38 |
| Infliximab+azathioprine/6MP+prednisone | Aminosalicylate | 0.23 | 2.57 | 0.00 | 35.77 |
| Tacrolimus | Antibiotic | 1.17 | 2.29 | 0.01 | 103.85 |
| Methotrexate+prednisone | Antibiotic | 2.90 | 1.80 | 0.08 | 99.30 |
| Azathioprine/6MP+prednisone | Antibiotic | 2.34 | 2.06 | 0.04 | 133.97 |
| Aminosalicylate+prednisone | Antibiotic | 7.22 | 2.64 | 0.04 | 1283.38 |
| Budesonide+prednisone | Antibiotic | 1.86 | 2.43 | 0.02 | 218.73 |
| MMF+prednisone | Antibiotic | 4.08 | 2.34 | 0.04 | 397.65 |
| Infliximab+azathioprine/6MP | Antibiotic | 1.09 | 1.26 | 0.09 | 12.78 |
| Azathioprine/6MP+aminosalicylate | Antibiotic | 1.33 | 2.36 | 0.01 | 136.82 |
| Natalizumab+infliximab | Antibiotic | 0.70 | 2.33 | 0.01 | 67.10 |
| Infliximab+azathioprine/6MP+prednisone | Antibiotic | 0.31 | 2.57 | 0.00 | 47.57 |
| Abbreviations: 6MP=6-mercaptopurine; MMF=mycophenolate mofetil | | | | | |
