# Supplementary material for: Inflammatory Bowel Disease (IBD) pharmacotherapy and the risk of serious infection: a systematic review and network meta-analysis
Source: BMC Gastroenterol. 2017 Apr 14;17:52. doi: 10.1186/s12876-017-0602-0 (PMC5391579; doi:10.1186/s12876-017-0602-0)
Supplement: Supplementary file 3 — Estimated odds of serious infection for treatment strategies compared to anti-tumor necrosis factor biologics. (DOCX 38 kb) [file 12876_2017_602_MOESM3_ESM.docx]

Supplementary Table 3: Estimated odds of serious infection for treatment strategies compared to anti-tumor necrosis factor biologics§

| **Treatment Strategy** | **Comparator** | **Odds Ratio** | **Standard Error** | **95% Confidence Interval** | |
| --- | --- | --- | --- | --- | --- |
| Methotrexate | Infliximab | 0.38 | 1.35 | 0.03 | 5.40 |
| Azathioprine/6MP | Infliximab | 1.05 | 0.47 | 0.42 | 2.63 |
| Prednisone | Infliximab | 1.41 | 0.75 | 0.32 | 6.13 |
| Budesonide | Infliximab | 1.46 | 1.60 | 0.06 | 33.90 |
| Aminosalicylate | Infliximab | 1.01 | 1.38 | 0.07 | 15.13 |
| Antibiotic | Infliximab | 0.75 | 1.16 | 0.08 | 7.29 |
| Tacrolimus | Infliximab | 0.87 | 2.07 | 0.02 | 50.35 |
| Methotrexate+prednisone | Infliximab | 2.16 | 1.39 | 0.14 | 32.92 |
| Azathioprine/6MP+prednisone | Infliximab | 1.74 | 1.72 | 0.06 | 50.37 |
| Aminosalicylate+prednisone | Infliximab | 5.38 | 2.38 | 0.05 | 571.50 |
| Budesonide+prednisone | Infliximab | 1.39 | 2.14 | 0.02 | 92.61 |
| MMF+prednisone | Infliximab | 3.04 | 2.03 | 0.06 | 163.96 |
| Azathioprine/6MP+aminosalicylate | Infliximab | 0.99 | 2.07 | 0.02 | 57.08 |
| Natalizumab+infliximab | Infliximab | 0.52 | 2.01 | 0.01 | 27.12 |
| Methotrexate | Adalimumab | 0.67 | 1.33 | 0.05 | 9.07 |
| Azathioprine/6MP | Adalimumab | 1.85 | 0.71 | 0.46 | 7.47 |
| Prednisone | Adalimumab | 2.49 | 0.93 | 0.41 | 15.27 |
| Budesonide | Adalimumab | 2.58 | 1.69 | 0.09 | 71.30 |
| Aminosalicylate | Adalimumab | 1.78 | 1.45 | 0.10 | 30.42 |
| Antibiotic | Adalimumab | 1.32 | 1.13 | 0.14 | 12.13 |
| Tacrolimus | Adalimumab | 1.54 | 2.05 | 0.03 | 86.01 |
| Methotrexate+prednisone | Adalimumab | 3.81 | 1.49 | 0.20 | 71.00 |
| Azathioprine/6MP+prednisone | Adalimumab | 3.08 | 1.80 | 0.09 | 104.79 |
| Aminosalicylate+prednisone | Adalimumab | 9.50 | 2.44 | 0.08 | 1137.24 |
| Budesonide+prednisone | Adalimumab | 2.45 | 2.21 | 0.03 | 186.68 |
| MMF+prednisone | Adalimumab | 5.37 | 2.11 | 0.09 | 332.76 |
| Infliximab+azathioprine/6MP | Adalimumab | 1.43 | 0.75 | 0.33 | 6.17 |
| Azathioprine/6MP+aminosalicylate | Adalimumab | 1.75 | 2.14 | 0.03 | 115.17 |
| Natalizumab+infliximab | Adalimumab | 0.93 | 2.09 | 0.02 | 56.03 |

**Supplementary Table 3, cont.: Estimated odds of serious infection for treatment strategies compared to anti-tumor necrosis factor biologics§**

| **Treatment Strategy** | **Comparator** | **Odds Ratio** | **Standard Error** | **95% Confidence Interval** | |
| --- | --- | --- | --- | --- | --- |
| Infliximab+azathioprine/6MP+prednisone | Adalimumab | 0.41 | 2.36 | 0.00 | 41.61 |
| Methotrexate | Certolizumab pegol | 0.22 | 1.37 | 0.01 | 3.23 |
| Azathioprine/6MP | Certolizumab pegol | 0.60 | 0.80 | 0.13 | 2.86 |
| Prednisone | Certolizumab pegol | 0.81 | 0.99 | 0.12 | 5.64 |
| Budesonide | Certolizumab pegol | 0.84 | 1.73 | 0.03 | 24.90 |
| Aminosalicylate | Certolizumab pegol | 0.58 | 1.49 | 0.03 | 10.75 |
| Antibiotic | Certolizumab pegol | 0.43 | 1.19 | 0.04 | 4.38 |
| Tacrolimus | Certolizumab pegol | 0.50 | 2.08 | 0.01 | 29.67 |
| Methotrexate+prednisone | Certolizumab pegol | 1.24 | 1.53 | 0.06 | 25.03 |
| Azathioprine/6MP+prednisone | Certolizumab pegol | 1.00 | 1.83 | 0.03 | 36.44 |
| Aminiosalicylate+prednisone | Certolizumab pegol | 3.09 | 2.47 | 0.02 | 388.64 |
| Budesonide+prednisone | Certolizumab pegol | 0.80 | 2.24 | 0.01 | 64.13 |
| MMF+prednisone | Certolizumab pegol | 1.75 | 2.13 | 0.03 | 114.62 |
| Infliximab+azathioprine/6MP | Certolizumab pegol | 0.46 | 0.83 | 0.09 | 2.34 |
| Azathioprine/6MP+aminosalicylate | Certolizumab pegol | 0.57 | 2.17 | 0.01 | 39.64 |
| Natalizumab+infliximab | Certolizumab pegol | 0.30 | 2.12 | 0.00 | 19.30 |
| Infliximab+azathioprine/6MP+prednisone | Certolizumab pegol | 0.13 | 2.38 | 0.00 | 14.25 |
| Methotrexate | Golimumab | 0.30 | 1.44 | 0.02 | 5.10 |
| Azathioprine/6MP | Golimumab | 0.83 | 0.91 | 0.14 | 4.92 |
| Prednisone | Golimumab | 1.12 | 1.08 | 0.13 | 9.35 |
| Budesonide | Golimumab | 1.16 | 1.78 | 0.04 | 38.35 |
| Aminosalicylate | Golimumab | 0.80 | 1.55 | 0.04 | 16.83 |
| Antibiotic | Golimumab | 0.59 | 1.26 | 0.05 | 7.06 |
| Tacrolimus | Golimumab | 0.69 | 2.13 | 0.01 | 44.90 |
| Methotrexate+prednisone | Golimumab | 1.72 | 1.59 | 0.08 | 39.05 |
| Azathioprine/6MP+prednisone | Golimumab | 1.39 | 1.88 | 0.03 | 55.79 |
| Aminiosalicylate+prednisone | Golimumab | 4.28 | 2.50 | 0.03 | 580.27 |

**Supplementary Table 3, cont.: Estimated odds of serious infection for treatment strategies compared to anti-tumor necrosis factor biologics§**

| **Treatment Strategy** | **Comparator** | **Odds Ratio** | **Standard Error** | **95% Confidence Interval** | |
| --- | --- | --- | --- | --- | --- |
| Budesonide+prednisone | Golimumab | 1.10 | 2.28 | 0.01 | 96.46 |
| MMF+prednisone | Golimumab | 2.42 | 2.18 | 0.03 | 173.09 |
| Infliximab+azathioprine/6MP | Golimumab | 0.64 | 0.93 | 0.10 | 4.01 |
| Azathioprine/6MP+aminosalicylate | Golimumab | 0.79 | 2.21 | 0.01 | 59.79 |
| Natalizumab+infliximab | Golimumab | 0.42 | 2.17 | 0.01 | 29.17 |
| Infliximab+azathioprine/6MP+prednisone | Golimumab | 0.18 | 2.42 | 0.00 | 21.32 |

| Abbreviations: 6MP=6-mercaptopurine; MMF=mycophenolate mofetil |  |
| --- | --- |
| §Other group comparisons can be found in Table 3 | |
