# Supplementary material for: Inflammatory Bowel Disease (IBD) pharmacotherapy and the risk of serious infection: a systematic review and network meta-analysis
Source: BMC Gastroenterol. 2017 Apr 14;17:52. doi: 10.1186/s12876-017-0602-0 (PMC5391579; doi:10.1186/s12876-017-0602-0)
Supplement: Supplementary file 4 — Estimated odds of serious infection for treatment strategies compared to Immunomodulators. (DOCX 30 kb) [file 12876_2017_602_MOESM4_ESM.docx]

Supplementary Table 4: Estimated odds of serious infection for treatment strategies compared to immunomodulators§

| **Treatment Strategy** | **Comparator** | **Odds Ratio** | **Standard Error** | **95% Confidence Interval** | |
| --- | --- | --- | --- | --- | --- |
| Prednisone | Methotrexate | 3.69 | 1.53 | 0.18 | 74.75 |
| Budesonide | Methotrexate | 3.83 | 2.09 | 0.06 | 229.95 |
| Aminosalicylate | Methotrexate | 2.64 | 1.90 | 0.06 | 108.59 |
| Antibiotic | Methotrexate | 1.95 | 1.67 | 0.07 | 51.32 |
| Tacrolimus | Methotrexate | 2.28 | 2.39 | 0.02 | 247.08 |
| Methotrexate+prednisone | Methotrexate | 5.65 | 1.93 | 0.13 | 248.43 |
| Azathioprine/6MP+prednisone | Methotrexate | 4.57 | 2.18 | 0.06 | 325.34 |
| Aminosalicylate+prednisone | Methotrexate | 14.09 | 2.73 | 0.07 | 2976.15 |
| Budesonide+prednisone | Methotrexate | 3.63 | 2.53 | 0.03 | 514.62 |
| MMF+prednisone | Methotrexate | 7.97 | 2.44 | 0.07 | 942.50 |
| Infliximab+azathioprine/6MP | Methotrexate | 2.12 | 1.43 | 0.13 | 35.20 |
| Azathioprine/6MP+aminosalicylate | Methotrexate | 2.60 | 2.46 | 0.02 | 323.57 |
| Natalizumab+infliximab | Methotrexate | 1.37 | 2.43 | 0.01 | 159.17 |
| Infliximab+azathioprine/6MP+prednisone | Methotrexate | 0.61 | 2.66 | 0.00 | 110.87 |
| Budesonide | Azathioprine/6MP | 1.40 | 1.61 | 0.06 | 32.63 |
| Aminosalicylate | Azathioprine/6MP | 0.96 | 1.38 | 0.06 | 14.37 |
| Antibiotic | Azathioprine/6MP | 0.71 | 1.24 | 0.06 | 8.04 |
| Tacrolimus | Azathioprine/6MP | 0.83 | 2.11 | 0.01 | 52.20 |
| Methotrexate+prednisone | Azathioprine/6MP | 2.06 | 1.40 | 0.13 | 31.74 |
| Azathioprine/6MP+prednisone | Azathioprine/6MP | 1.66 | 1.72 | 0.06 | 48.46 |
| Aminosalicylate+prednisone | Azathioprine/6MP | 5.13 | 2.38 | 0.05 | 548.49 |
| Budesonide+prednisone | Azathioprine/6MP | 1.32 | 2.15 | 0.02 | 88.93 |
| MMF+prednisone | Azathioprine/6MP | 2.90 | 2.04 | 0.05 | 157.53 |
| Azathioprine/6MP+aminosalicylate | Azathioprine/6MP | 0.95 | 2.01 | 0.02 | 48.96 |
| Natalizumab+infliximab | Azathioprine/6MP | 0.50 | 2.07 | 0.01 | 28.76 |
| Infliximab+azathioprine/6MP+prednisone | Azathioprine/6MP | 0.22 | 2.30 | 0.00 | 19.99 |
| Abbreviations: 6MP=6-mercaptopurine; MMF=mycophenolate mofetil | |  |  |  |  |
| §Other group comparisons can be found in Table 4 | |  |  |  |  |
