# Supplementary material for: Inflammatory Bowel Disease (IBD) pharmacotherapy and the risk of serious infection: a systematic review and network meta-analysis
Source: BMC Gastroenterol. 2017 Apr 14;17:52. doi: 10.1186/s12876-017-0602-0 (PMC5391579; doi:10.1186/s12876-017-0602-0)
Supplement: Supplementary file 5 — Estimated odds of serious infection for treatment strategies compared to other immunosuppressants. (DOCX 33 kb) [file 12876_2017_602_MOESM5_ESM.docx]

Supplementary Table 5: Estimated odds of serious infection for treatment strategies compared to other immunosuppressants

| **Treatment Strategy** | **Comparator** | **Odds Ratio** | **Standard Error** | **95% Confidence Interval** | |
| --- | --- | --- | --- | --- | --- |
| Budesonide | Prednisone | 1.04 | 1.42 | 0.06 | 16.70 |
| Aminosalicylate | Prednisone | 0.71 | 1.39 | 0.05 | 10.84 |
| Antibiotic | Prednisone | 0.53 | 1.37 | 0.04 | 7.77 |
| Tacrolimus | Prednisone | 0.62 | 2.19 | 0.01 | 45.52 |
| Methotrexate+prednisone | Prednisone | 1.53 | 1.17 | 0.15 | 15.19 |
| Azathioprine/6MP+prednisone | Prednisone | 1.24 | 1.54 | 0.06 | 25.50 |
| Aminosalicylate+prednisone | Prednisone | 3.82 | 2.26 | 0.05 | 319.84 |
| Budesonide+prednisone | Prednisone | 0.98 | 2.01 | 0.02 | 50.38 |
| MMF+prednisone | Prednisone | 2.16 | 1.89 | 0.05 | 87.89 |
| Infliximab+Azathioprine/6MP | Prednisone | 0.57 | 0.61 | 0.17 | 1.90 |
| Azathioprine/6MP+aminosalicylate | Prednisone | 0.70 | 2.15 | 0.01 | 47.78 |
| Natalizumab+infliximab | Prednisone | 0.37 | 2.15 | 0.01 | 25.07 |
| Infliximab+Azathioprine/6MP+prednisone | Prednisone | 0.16 | 2.17 | 0.00 | 11.54 |
| Aminosalicylate | Budesonide | 0.69 | 1.98 | 0.01 | 33.60 |
| Antibiotic | Budesonide | 0.51 | 1.97 | 0.01 | 24.31 |
| Tacrolimus | Budesonide | 0.60 | 2.61 | 0.00 | 99.52 |
| Methotrexate+prednisone | Budesonide | 1.47 | 1.84 | 0.04 | 54.17 |
| Azathioprine/6MP+prednisone | Budesonide | 1.19 | 2.10 | 0.02 | 72.48 |
| Aminosalicylate+prednisone | Budesonide | 3.68 | 2.67 | 0.02 | 685.33 |
| Budesonide+prednisone | Budesonide | 0.95 | 2.46 | 0.01 | 117.21 |
| MMF+prednisone | Budesonide | 2.08 | 2.36 | 0.02 | 213.66 |
| Infliximab+Azathioprine/6MP | Budesonide | 0.55 | 1.54 | 0.03 | 11.38 |
| Azathioprine/6MP+aminosalicylate | Budesonide | 0.68 | 2.58 | 0.00 | 105.84 |
| Natalizumab+infliximab | Budesonide | 0.36 | 2.57 | 0.00 | 55.60 |
| Infliximab+Azathioprine/6MP+prednisone | Budesonide | 0.16 | 2.59 | 0.00 | 25.44 |
| Abbreviations: 6MP=6-mercaptopurine; MMF=mycophenolate mofetil | | |  |  |  |

**Supplementary Table 5, cont.: Estimated odds of serious infection for treatment strategies compared to other immunosuppressants**

| **Treatment Strategy** | **Comparator** | **Odds Ratio** | **Standard Error** | **95% Confidence Interval** | |
| --- | --- | --- | --- | --- | --- |
| Methotrexate+prednisone | Tacrolimus | 2.48 | 2.49 | 0.02 | 324.07 |
| Azathioprine/6MP+prednisone | Tacrolimus | 2.00 | 2.68 | 0.01 | 384.33 |
| Aminosalicylate+prednisone | Tacrolimus | 6.18 | 3.15 | 0.01 | 2959.71 |
| Budesonide+prednisone | Tacrolimus | 1.59 | 2.97 | 0.00 | 541.43 |
| MMF+prednisone | Tacrolimus | 3.49 | 2.90 | 0.01 | 1019.73 |
| Infliximab+Azathioprine/6MP | Tacrolimus | 0.93 | 2.12 | 0.01 | 59.75 |
| Azathioprine/6MP+aminosalicylate | Tacrolimus | 1.14 | 2.92 | 0.00 | 347.20 |
| Natalizumab+infliximab | Tacrolimus | 0.60 | 2.89 | 0.00 | 172.75 |
| Infliximab+Azathioprine/6MP+prednisone | Tacrolimus | 0.27 | 3.08 | 0.00 | 112.45 |
| Abbreviations: 6MP=6-mercaptopurine; MMF=mycophenolate mofetil | | |  |  |  |
