# Supplementary material for: Inflammatory Bowel Disease (IBD) pharmacotherapy and the risk of serious infection: a systematic review and network meta-analysis
Source: BMC Gastroenterol. 2017 Apr 14;17:52. doi: 10.1186/s12876-017-0602-0 (PMC5391579; doi:10.1186/s12876-017-0602-0)
Supplement: Supplementary file 6 — Estimated odds of serious infection for treatment strategies compared to other biologics. (DOCX 35 kb) [file 12876_2017_602_MOESM6_ESM.docx]

Supplementary Table 6: Estimated odds of serious infection for treatment strategies compared to other biologics§

| **Treatment Strategy** | **Comparator** | **Odds Ratio** | **Standard Error** | **95% Confidence Interval** | |
| --- | --- | --- | --- | --- | --- |
| Methotrexate | Natalizumab | 0.65 | 1.34 | 0.05 | 8.97 |
| Azathioprine/6MP | Natalizumab | 1.79 | 0.73 | 0.43 | 7.52 |
| Prednisone | Natalizumab | 2.41 | 0.94 | 0.38 | 15.23 |
| Budesonide | Natalizumab | 2.50 | 1.70 | 0.09 | 70.20 |
| Aminosalicylate | Natalizumab | 1.72 | 1.46 | 0.10 | 30.04 |
| Antibiotic | Natalizumab | 1.27 | 1.15 | 0.14 | 12.03 |
| Tacrolimus | Natalizumab | 1.49 | 2.06 | 0.03 | 84.45 |
| Methotrexate+prednisone | Natalizumab | 3.69 | 1.50 | 0.19 | 70.06 |
| Azathioprine/6MP+prednisone | Natalizumab | 2.98 | 1.81 | 0.09 | 103.08 |
| Aminosalicylate+prednisone | Natalizumab | 9.20 | 2.45 | 0.08 | 1114.31 |
| Budesonide+prednisone | Natalizumab | 2.37 | 2.22 | 0.03 | 183.13 |
| MMF+prednisone | Natalizumab | 5.21 | 2.11 | 0.08 | 326.63 |
| Infliximab+azathioprine/6MP | Natalizumab | 1.38 | 0.76 | 0.31 | 6.19 |
| Azathioprine/6MP+aminosalicylate | Natalizumab | 1.70 | 2.14 | 0.03 | 113.03 |
| Natalizumab+infliximab | Natalizumab | 0.90 | 2.10 | 0.01 | 55.00 |
| Infliximab+azathioprine/6MP+prednisone | Natalizumab | 0.40 | 2.36 | 0.00 | 40.79 |
| Methotrexate | Ustekinumab | 1.29 | 1.37 | 0.09 | 18.72 |
| Azathioprine/6MP | Ustekinumab | 3.54 | 0.78 | 0.76 | 16.41 |
| Prednisone | Ustekinumab | 4.76 | 0.98 | 0.70 | 32.54 |
| Budesonide | Ustekinumab | 4.94 | 1.72 | 0.17 | 144.78 |
| Aminosalicylate | Ustekinumab | 3.40 | 1.48 | 0.19 | 62.40 |
| Antibiotic | Ustekinumab | 2.51 | 1.18 | 0.25 | 25.35 |
| Tacrolimus | Ustekinumab | 2.94 | 2.08 | 0.05 | 172.81 |
| Methotrexate+prednisone | Ustekinumab | 7.28 | 1.53 | 0.36 | 145.33 |
| Azathioprine/6MP+prednisone | Ustekinumab | 5.88 | 1.83 | 0.16 | 212.01 |
| Aminosalicylate+prednisone | Ustekinumab | 18.15 | 2.46 | 0.15 | 2266.86 |

**Supplementary Table 6, cont.: Estimated odds of serious infection for treatment strategies compared to other biologics§**

| **Treatment Strategy** | **Comparator** | **Odds Ratio** | **Standard Error** | **95% Confidence Interval** | |
| --- | --- | --- | --- | --- | --- |
| Budesonide+prednisone | Ustekinumab | 4.68 | 2.24 | 0.06 | 373.75 |
| MMF+prednisone | Ustekinumab | 10.26 | 2.13 | 0.16 | 667.76 |
| Infliximab+azathioprine/6MP | Ustekinumab | 2.73 | 0.81 | 0.55 | 13.46 |
| Azathioprine/6MP+aminosalicylate | Ustekinumab | 3.34 | 2.16 | 0.05 | 230.95 |
| Natalizumab+infliximab | Ustekinumab | 1.77 | 2.12 | 0.03 | 112.46 |
| Infliximab+azathioprine/6MP+prednisone | Ustekinumab | 0.78 | 2.38 | 0.01 | 83.07 |
| Methotrexate | Vedolizumab | 0.69 | 1.33 | 0.05 | 9.36 |
| Azathioprine/6MP | Vedolizumab | 1.89 | 0.72 | 0.46 | 7.77 |
| Prednisone | Vedolizumab | 2.55 | 0.93 | 0.41 | 15.82 |
| Budesonide | Vedolizumab | 2.64 | 1.70 | 0.10 | 73.46 |
| Aminosalicylate | Vedolizumab | 1.82 | 1.45 | 0.11 | 31.38 |
| Antibiotic | Vedolizumab | 1.35 | 1.14 | 0.14 | 12.54 |
| Tacrolimus | Vedolizumab | 1.57 | 2.06 | 0.03 | 88.50 |
| Methotrexate+prednisone | Vedolizumab | 3.90 | 1.50 | 0.21 | 73.22 |
| Azathioprine/6MP+prednisone | Vedolizumab | 3.15 | 1.80 | 0.09 | 107.91 |
| Aminosalicylate+prednisone | Vedolizumab | 9.72 | 2.44 | 0.08 | 1169.11 |
| Budesonide+prednisone | Vedolizumab | 2.50 | 2.21 | 0.03 | 192.01 |
| MMF+prednisone | Vedolizumab | 5.50 | 2.11 | 0.09 | 342.35 |
| Infliximab+azathioprine/6MP | Vedolizumab | 1.46 | 0.75 | 0.33 | 6.41 |
| Azathioprine/6MP+aminosalicylate | Vedolizumab | 1.79 | 2.14 | 0.03 | 118.48 |
| Natalizumab+infliximab | Vedolizumab | 0.95 | 2.10 | 0.02 | 57.64 |
| Infliximab+azathioprine/6MP+prednisone | Vedolizumab | 0.42 | 2.36 | 0.00 | 42.79 |
| Abbreviations: 6MP=6-mercaptopurine; MMF=mycophenolate mofetil | |  |  |  |  |
| §Other group comparisons can be found in Table 4 | |  |  |  |  |
