# Supplementary material for: Inflammatory Bowel Disease (IBD) pharmacotherapy and the risk of serious infection: a systematic review and network meta-analysis
Source: BMC Gastroenterol. 2017 Apr 14;17:52. doi: 10.1186/s12876-017-0602-0 (PMC5391579; doi:10.1186/s12876-017-0602-0)
Supplement: Supplementary file 7 — Estimated odds of serious infection for treatment strategies compared to combination therapies. (DOCX 33 kb) [file 12876_2017_602_MOESM7_ESM.docx]

Supplementary Table 7: Estimated odds of serious infection for treatment strategies compared to combination therapies

| **Treatment Strategy** | **Comparator** | **Odds Ratio** | **Standard Error** | **95% Confidence Interval** | |
| --- | --- | --- | --- | --- | --- |
| Azathioprine/6MP+prednisone | Methotrexate+prednisone | 0.81 | 1.54 | 0.04 | 16.64 |
| Aminosalicylate+prednisone | Methotrexate+prednisone | 2.49 | 2.26 | 0.03 | 208.77 |
| Budesonide+prednisone | Methotrexate+prednisone | 0.64 | 2.32 | 0.01 | 61.21 |
| MMF+prednisone | Methotrexate+prednisone | 1.41 | 1.89 | 0.03 | 57.36 |
| Infliximab+azathioprine/6MP | Methotrexate+prednisone | 0.38 | 1.32 | 0.03 | 4.99 |
| Azathioprine/6MP+aminosalicylate | Methotrexate+prednisone | 0.46 | 2.45 | 0.00 | 55.97 |
| Natalizumab+infliximab | Methotrexate+prednisone | 0.24 | 2.45 | 0.00 | 29.39 |
| Infliximab+azathioprine/6MP+prednisone | Methotrexate+prednisone | 0.11 | 2.17 | 0.00 | 7.54 |
| Aminosalicylate+prednisone | Azathioprine/6MP+prednisone | 3.08 | 1.65 | 0.12 | 78.27 |
| Budesonide+prednisone | Azathioprine/6MP+prednisone | 0.79 | 2.53 | 0.01 | 113.94 |
| MMF+prednisone | Azathioprine/6MP+prednisone | 1.74 | 1.09 | 0.21 | 14.84 |
| Infliximab+azathioprine/6MP | Azathioprine/6MP+prednisone | 0.46 | 1.66 | 0.02 | 12.00 |
| Azathioprine/6MP+aminosalicylate | Azathioprine/6MP+prednisone | 0.57 | 2.65 | 0.00 | 102.17 |
| Natalizumab+infliximab | Azathioprine/6MP+prednisone | 0.30 | 2.65 | 0.00 | 53.68 |
| Infliximab+azathioprine/6MP+prednisone | Azathioprine/6MP+prednisone | 0.13 | 1.52 | 0.01 | 2.63 |
| Budesonide+prednisone | Aminosalicylate+prednisone | 0.26 | 3.02 | 0.00 | 96.49 |
| MMF+prednisone | Aminosalicylate+prednisone | 0.57 | 1.98 | 0.01 | 27.34 |
| Infliximab+azathioprine/6MP | Aminosalicylate+prednisone | 0.15 | 2.34 | 0.00 | 14.77 |
| Azathioprine/6MP+aminosalicylate | Aminosalicylate+prednisone | 0.18 | 3.12 | 0.00 | 83.51 |
| Natalizumab+infliximab | Aminosalicylate+prednisone | 0.10 | 3.12 | 0.00 | 43.91 |
| Infliximab+azathioprine/6MP+prednisone | Aminosalicylate+prednisone | 0.04 | 2.25 | 0.00 | 3.52 |
| MMF+prednisone | Budesonide+prednisone | 2.19 | 2.76 | 0.01 | 489.43 |
| Infliximab+azathioprine/6MP | Budesonide+prednisone | 0.58 | 2.10 | 0.01 | 35.71 |
| Azathioprine/6MP+aminosalicylate | Budesonide+prednisone | 0.71 | 2.94 | 0.00 | 229.24 |
| Natalizumab+infliximab | Budesonide+prednisone | 0.38 | 2.94 | 0.00 | 120.52 |
| Infliximab+azathioprine/6MP+prednisone | Budesonide+prednisone | 0.17 | 2.96 | 0.00 | 54.91 |

**Supplementary Table 7, cont.: Estimated odds of serious infection for treatment strategies compared to combination therapies**

| **Treatment Strategy** | **Comparator** | **Odds Ratio** | **Standard Error** | **95% Confidence Interval** | |
| --- | --- | --- | --- | --- | --- |
| Infliximab+azathioprine/6MP | MMF+prednisone | 0.27 | 1.99 | 0.01 | 13.07 |
| Azathioprine/6MP+aminosalicylate | MMF+prednisone | 0.33 | 2.87 | 0.00 | 89.51 |
| Natalizumab+infliximab | MMF+prednisone | 0.17 | 2.86 | 0.00 | 47.05 |
| Infliximab+azathioprine/6MP+prednisone | MMF+prednisone | 0.08 | 1.87 | 0.00 | 3.00 |
| Azathioprine/6MP+aminosalicylate | Infliximab+azathioprine/6MP | 1.22 | 2.08 | 0.02 | 71.56 |
| Natalizumab+infliximab | Infliximab+azathioprine/6MP | 0.65 | 2.08 | 0.01 | 37.86 |
| Infliximab+azathioprine/6MP+prednisone | Infliximab+azathioprine/6MP | 0.29 | 2.25 | 0.00 | 23.70 |
| Natalizumab+infliximab | Azathioprine/6MP+aminosalicylate | 0.53 | 2.89 | 0.00 | 151.42 |
| Infliximab+azathioprine/6MP+prednisone | Azathioprine/6MP+aminosalicylate | 0.23 | 3.06 | 0.00 | 93.36 |
| Infliximab+azathioprine/6MP+prednisone | Natalizumab+infliximab | 0.44 | 3.05 | 0.00 | 175.71 |
| Abbreviations: 6MP=6-mercaptopurine; MMF=mycophenolate mofetil | |  |  |  |  |
